# *DRH1*, a p68-related RNA helicase gene*,* is required for chromosome breakage in *Tetrahymena*

**DOI:** 10.1242/bio.021576

**Published:** 2016-10-28

**Authors:** Stephen L. McDaniel, Erica Zweifel, Peter K. W. Harris, Meng-Chao Yao, Eric S. Cole, Douglas L. Chalker

**Affiliations:** 1 Department of Biology, Washington University in St. Louis, St Louis, MO 63130, USA; 2 Biology Department, St. Olaf College, 1520 St. Olaf Avenue, Northfield, MN 55057, USA; 3 Institute of Molecular Biology, Academia Sinica, Nankang, Taipei 11529, Taiwan

**Keywords:** Ciliate, Genome rearrangements, DEAD-box RNA Helicase, p68, Chromosome, Meiosis

## Abstract

The p68 DEAD box helicases comprise a widely conserved protein family involved in a large range of biological processes including transcription, splicing and translation. The genome of the ciliate *Tetrahymena thermophile* encodes two p68-like helicases, Drh1p and Lia2p. We show that *DRH1* is essential for growth and completion of development. In growing cells, Drh1p is excluded from the nucleus and accumulates near cortical basal bodies. In contrast, during sexual reproduction, this protein localizes to meiotic micronuclei, initially in punctate foci in regions where centromeres and telomeres are known to reside and later in post-zygotic differentiating somatic macronuclei. Differentiation of the macronuclear genome involves extensive DNA rearrangements including fragmentation of the five pairs of germline-derived chromosomes into 180 chromosomal sub-fragments that are stabilized by *de novo* telomere deletion. In addition, thousands of internal eliminated sequences (IESs) are excised from loci dispersed throughout the genome. Strains with *DRH1* deleted from the germline nuclei, which do not express the protein during post-zygotic development, fail to fragment the developing macronuclear chromosomes. IES excision still occurs in the absence of *DRH1* zygotic expression; thus, Drh1p is the first protein found to be specifically required for chromosome breakage but not DNA elimination.

## Abstract (precis)

**Summary:** The p68-related Drh1protein is essential for both growth and development of the ciliate *Tetrahymena thermophila*. It localizes to meiotic nuclei and is required for chromosome breakage of developing somatic chromosomes.

## INTRODUCTION

Like all ciliated protozoans, *Tetrahymena thermophila* contains two functionally distinct types of nuclei: a somatic macronucleus and a germline micronucleus. When *Tetrahymena* cells mate, they undergo a predictable genetic program (reviewed by Cole and Sugai, 2012) of meiosis, cross-fertilization (exchange of gametic nuclei), karyogamy (fusion of gametic nuclei), DNA replication, nuclear division, and new macronuclear genome differentiation. Nuclear differentiation involves extensive genome remodeling encompassing two processes: massive DNA elimination and chromosome fragmentation. DNA elimination removes nearly one third of the germline-derived genome from the newly forming somatic genome. The thousands of DNA segments removed, termed internal eliminated sequences (IESs), are composed largely of A+T-rich non-coding sequences, transposable elements, and other repetitive DNA (Chalker and Yao, 2011). The resulting chromosomal breaks are repaired by non-homologous end joining (Lin et al., 2012). This pathway generates a gene-enriched genome specialized for its somatic role in growing progeny.

Chromosome fragmentation occurs when the five pairs of germline-derived chromosomes are processed into ∼180 macronuclear chromosomes (Hamilton et al., 2005); this reduction in chromosome size is thought to be important to facilitate the amitotic division of the macronucleus in growing cells. The loci at which genome fragmentation occurs contain a highly conserved 15 bp chromosomal breakage sequence (CBS), which is necessary and sufficient for this processing (Fan and Yao, 1996, 2000). This sequence is entirely removed from the somatic chromosomes, and new telomeres are added within 25 bp of the former position of the CBS.

The process and many of the proteins involved in IES elimination are well characterized. First, non-genic transcripts produced during meiosis are processed by Dicer-like 1 (Dcl1p) into ∼30 nt scanRNAs (scnRNAs). These scnRNAs bind the *Tetrahymena* Piwi1 (Twi1p)-interacting protein and mark the sequences to be eliminated in the differentiating macronucleus (Mochizuki et al., 2002; Chalker and Yao, 2001; Malone et al., 2005; Mochizuki and Gorovsky, 2005). Next, Twi1p-bound scnRNAs target histone H3 lysine 9 (K9) and K27 methylation to homologous sequences in the developing macronucleus. Finally, the IESs are excised by the domesticated transposase Tpb2p (Taverna et al., 2002; Liu et al., 2007; Cheng et al., 2010). The RNA helicase Ema1p is also essential for the histone methylation that leads to IES elimination. Ema1p is thought to accomplish this by stimulating base-pairing between Twi1p-bound scnRNAs and noncoding transcripts in both the parental and developing somatic nuclei.

Unlike the DNA elimination pathway, the proteins that recognize and cleave at the CBS and eventually recruit telomerase remain to be discovered. Deletion of genes encoding components (e.g. *DCL1*) of the DNA elimination machinery block chromosome fragmentation, possibly indicating overlap in these pathways. Alternatively, the developmental arrest caused by these mutations may occur before chromosomal fragmentation initiates (Malone et al., 2005). The developmental timing and interdependence between these two types of rearrangements remains to be determined. To identify the molecular machinery that performs IES elimination and chromosome breakage, we have focused on proteins that have induced expression during development, localize to nuclei where these processes occur, and/or share homology to proteins previously linked to these events (Yao et al., 2007; Matsuda et al., 2010). The importance of homologous RNAs in DNA rearrangements, the known role for Ema1p in DNA rearrangements (Aronica et al., 2008), and the abundance of RNA helicases encoded within the *Tetrahymena* genome led us to further investigate members of this family of proteins for possible roles in genome reorganization.

RNA helicases participate in the majority of biological processes involving RNA, including transcription, processing, and degradation. In these processes, RNA helicases unwind secondary structures and participate in assembly/disassembly of ribonucleoprotein complexes and other mechanisms requiring RNA structural manipulation (Fuller-Pace, 2006; Linder, 2006; Cordin et al., 2006). Ema1p belongs to the DExD/H box family of helicases, which includes the well-known DEAD box proteins. Some DExD/H proteins are thought to act in single pathways, whereas others participate in multiple biological processes (Linder and Jankowsky, 2011). The human, yeast, and *Tetrahymena* genomes encode 37, 26, and 45 (*DRH1-45*) DExD/H box family members, respectively (Fairman-Williams et al., 2010). All evidence indicates that these proteins have undergone extensive diversification during their evolution.

Although called helicases, how, or even whether, specific DExD/H box proteins unwind RNAs is uncertain (Jankowsky, 2011). They clearly can alter RNA structure in ways that lead to RNA unwinding or assist in RNA annealing. However, some family members have been implicated in processes for which their action may not be dependent on RNA. For example, the p68 (DDX5) and related p72 (DDX17) proteins have roles in transcriptional regulation that appear to be independent from their helicase activity (Endoh et al., 1999; Watanabe et al., 2001). Nevertheless, these same proteins participate in pre-mRNA, rRNA, and microRNA processing (Fuller-Pace and Moore, 2011). How these proteins can participate in such a diverse array of cellular events is not well understood.

In previous work, we identified the DEAD box helicase gene *LIA2* in a screen for developmentally expressed proteins that localize within developing somatic nuclei (Yao et al., 2007). The function of this protein is unknown, but preliminary data indicated that this protein is not essential for growth (Yao et al., 2007; Fass et al., 2011). Here, we have investigated the *Tetrahymena* protein most closely related to the protein encoded by *LIA2*, the p68-like DEAD box RNA helicase Drh1p. We report that its zygotic expression is essential for chromosome fragmentation, but not DNA elimination. This finding makes Drh1p the first protein known to be required specifically for chromosome breakage.

## RESULTS

### *Tetrahymena* encodes two putative p68-like RNA helicases

We previously identified *LIA2* (*DRH3*) in a screen for proteins that localize within developing macronuclei. We disrupted the gene by recombining the *neo3* (*MTT1-neo*) selectable marker (which confers paromomycin resistance to *Tetrahymena* cells) (Shang et al., 2002) into the middle of the coding region (Fig. S1A) but did not detect any obvious phenotypes (Yao et al., 2007; Fass et al., 2011). During subsequent work, we observed that mutant cells divided more slowly than wild-type cells when grown in stationary cultures (data not shown), but this growth difference was not apparent when cells were grown in flasks with shaking (Fig. S1B). Thus, any growth impairment proved too weak to use to help uncover processes in which the Lia2 protein (Lia2p) might act.

**Fig. 1.**
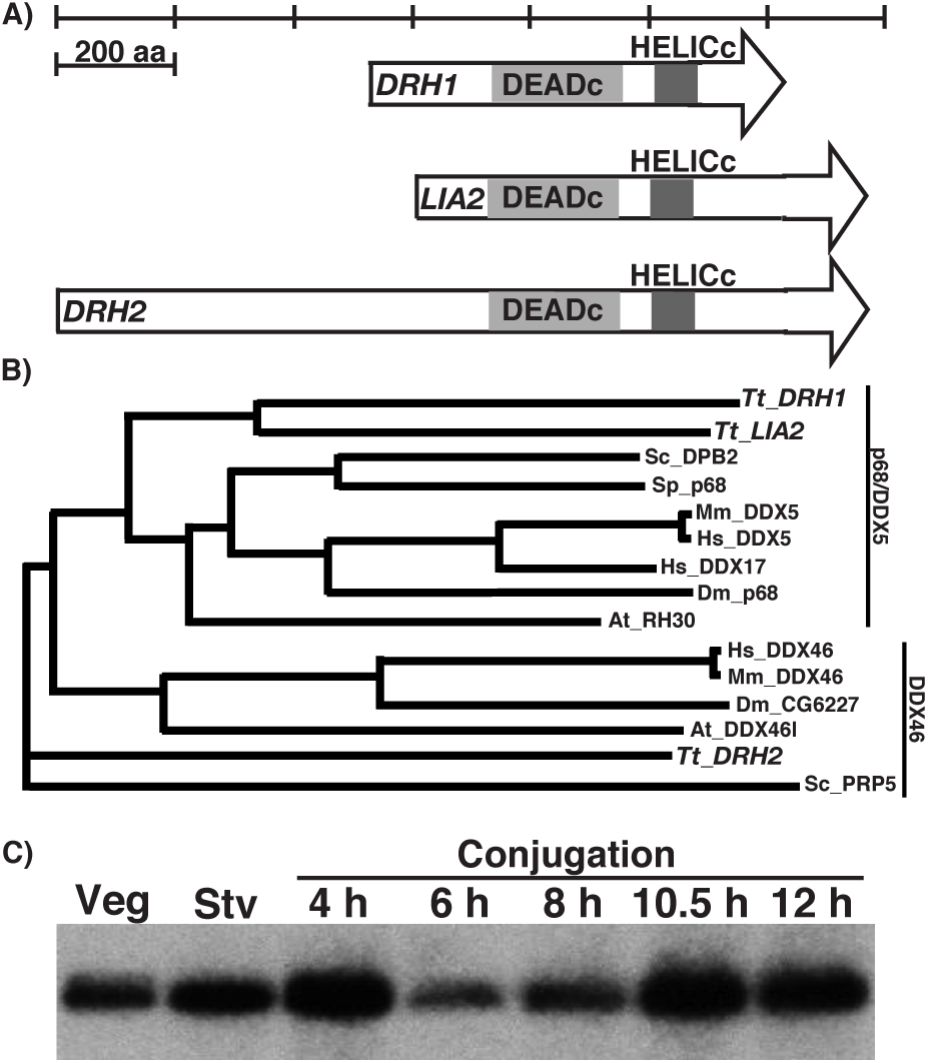
**The conserved Drh1p helicase is expressed during *Tetrahymena* growth and development.** (A) The three DEAD-box helicase proteins encoded in the genome most similar to the p68/DDX5 family are illustrated below a scale bar. The conserved DEADc and HELICc domains are aligned. (B) A phyllogram generated by multiple sequence alignment of p68- and DDX46-related protein sequences. (C) Northern blot analysis of *DRH1* expression during vegetative growth (veg), starvation (Stv) and the indicated time point post-mixing to initiate conjugation.

*LIA2* is one of 45 *Tetrahymena* genes (*DRH1*-*DRH45*) encoding putative DExD/H box RNA helicases and is highly similar to a second gene, *DRH1* (TTHERM_00190830). Both genes encode putative homologs in the p68/DDX5 family of helicases, although the similarity is confined to the ∼430 amino acid helicase domains (DEADc and HELICc in Fig. 1A; see Fig. S2). *LIA2* and *DRH1* are apparent paralogs as they are more similar to each other than to any other sequence in the Genbank non-redundant database (Fig. 1B). Reciprocal BLAST searches and multi-sequence alignments showed that both genes cluster with p68 proteins, whereas the *Tetrahymena* protein (encoded by *DRH2*, Ttherm_00420420) possessing the next greatest similarity to p68 proteins clusters with the DDX46 family of helicases (Fig. 1B).

Both *LIA2* and *DRH1* are expressed throughout the life cycle. Both genes are expressed at moderately high levels during growth and starvation (a condition of nutrient deprivation that readies cells for conjugation when cells of complementary mating types are mixed) and are upregulated during conjugation. *LIA2* mRNA accumulation increases steadily at the beginning of post-zygotic development (6 h into conjugation) (Yao et al., 2007), whereas *DRH1* is upregulated starting in pre-zygotic development (4 h; Fig. 1C), when meiosis occurs. *DRH1* mRNA levels decrease during the early post-zygotic stages of development (6 to 8 h post-pairing) and rise again by 10.5 h, which corresponds to the onset of DNA rearrangements that remodel the somatic genome within differentiating macronuclei. These differences in sequence and expression suggest that these two paralogs have diverged somewhat in function.

### Drh1p shows dynamic organization in nuclei during development

**Fig. 2.**
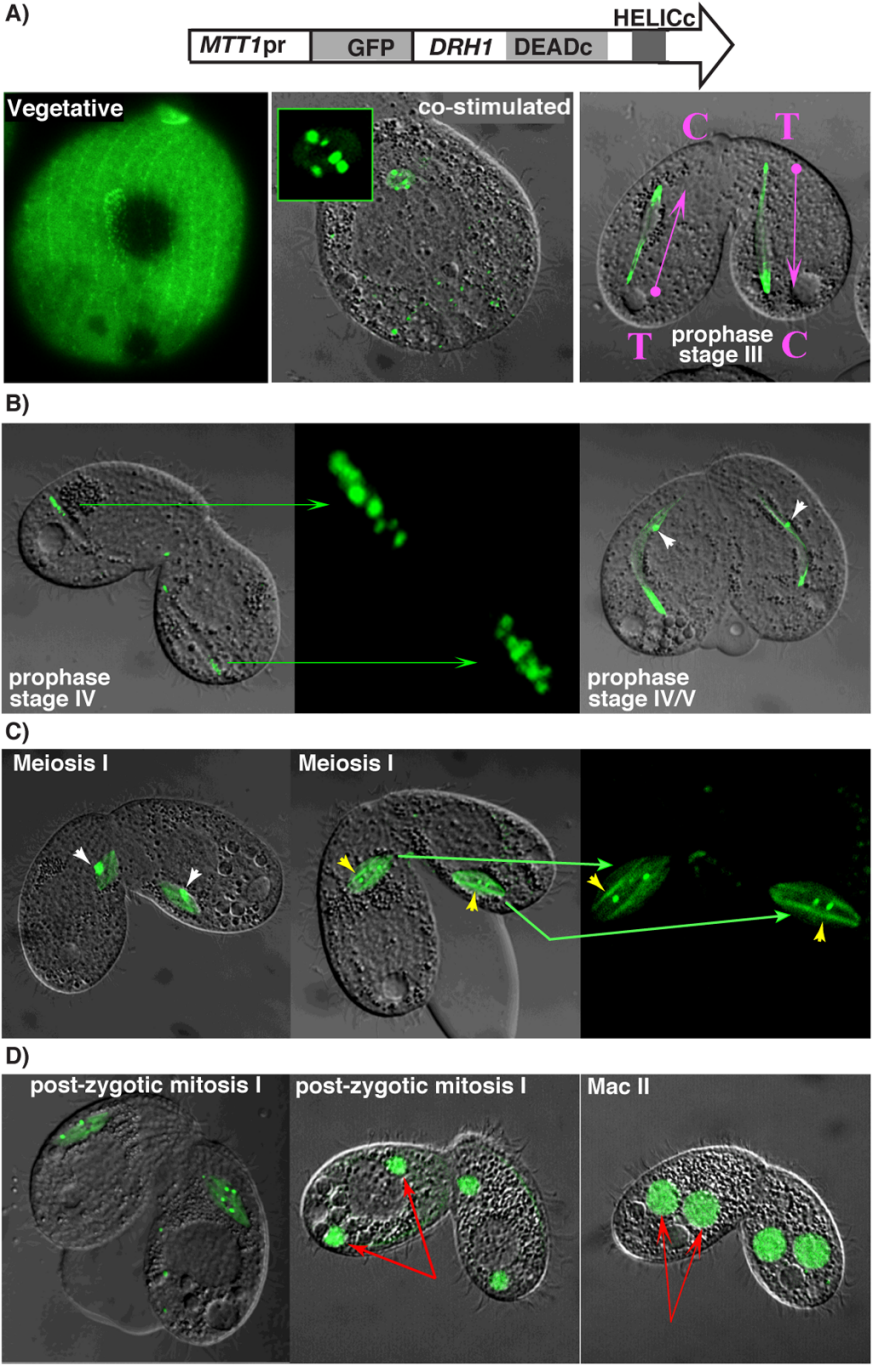
**Drh1p** **exhibits diverse localization during growth and development.** The localization of GFP-Drh1p, expressed from the inducible transgene shown, was visualized by fluorescence confocal microscopy at the indicated stages of growth. (A) Vegetative cell in growth media through early mating; green shows expression of protein encoded by *ΔDRH1*::GFP-DRH1. Inset of center panel highlights punctate localization in micronuclei upon co-stimulation. For the pair in prophase, stage III, magenta arrows point from the telomere (T)-containing apical end of ‘egg-stage’ micronuclei towards the enlarged end containing centromeres (C). (B) Late prophase (stage IV), center panel shows enlarged image of fluorescence highlighting the punctate localization at centromeric end. White arrowheads indicate GFP-Drh1p foci in the ‘head’ (telomere end) of micronuclei that remain into early Meiosis I shown in (C). Yellow arrowheads point to GFP enrichment in first meiotic spindle filaments more easily observed in the rightmost panel, white arrows as in B. Enlarged sections from middle panel shown in right panel, green arrows highlight areas of GFP-Drh1p expression. (D) Localization of GFP-Drh1p during post-zygotic development. Red arrows indicate differentiating macronuclei.

The p68 RNA helicases, including Lia2p, are primarily nuclear-localized proteins that have been implicated in a variety of cellular processes (Fuller-Pace and Moore, 2011). To gain insight into functions of Drh1p, we examined the sub-cellular localization of a green fluorescent protein (GFP)-*DRH1* fusion protein expressed throughout the *Tetrahymena* life cycle from the cadmium-inducible *MTT1* promoter. In vegetatively growing cells, GFP-Drh1p accumulated in the cytoplasm but was largely undetectable in nuclei, which is not what we expected. The protein was somewhat enriched at the cell cortex, possibly near basal bodies (Fig. 2A; Fig. S3), but the significance of this cortical localization is not known.

Shortly after mixing pre-starved populations of complementary mating types, GFP-Drh1p strongly accumulated within germline micronuclei (Fig. 2A). Protein import occurred rapidly, even prior to cell pairing, during the period of co-stimulation. The GFP fluorescence within micronuclei was initially observed in about five punctate spots (inset, Fig. 2A). *Tetrahymena* micronuclei contain five pairs of germline chromosomes that undergo recombination during late meiotic prophase. It is possible that centromeres of homologous chromosomes align even at this earlier stage. If so, the ten centromeric regions would appear as five independent foci such as we observed here. We speculate that the five GFP-decorated foci are the centromeric regions of the paired micronuclear chromosomes.

Shortly after cell pairing, micronuclei elongate to create the ‘crescent’ micronucleus, which reaches its maximum extended length at prophase stage IV (Fig. 2B). During stage III, the elongating micronuclei have a narrow ‘head’ and a bulb-shaped trunk. Chromosomes occupy a non-random configuration within this structure, with the telomeres concentrated at the tip of the head and centromeres positioned near the base of the trunk (Loidl and Scherthan, 2004; Cervantes et al., 2006). At this stage, GFP-Drh1p was enriched at both ends of crescent micronuclei, concentrated at the tip of the head (the telomere end) and exhibiting a more dispersed granular appearance near the apical end of the trunk (centromeres). Approximately ten GFP-labeled aggregates could be counted in the trunk region of each crescent micronucleus, which is consistent with the number of centromeres at this stage. As the crescent micronucleus begins to condense (the transition between prophase stages IV and V), the GFP fluorescence at both ends became more dispersed throughout the crescent (Fig. S4). Other cells at this stage showed diffuse Drh1p localization at both ends and a single large aggregate near the centromeric end. This dynamic localization near telomeres and centromeres is consistent with a role of Drh1p in meiotic chromosome function.

Entering metaphase of meiosis I (note: *Tetrahymena* undergoes closed meiosis without nuclear envelope breakdown), GFP-Drh1p was localized over both fibers and distinct foci within the spindle apparatus (Fig. 2C). We frequently observed a single dense focus located mid-spindle that later appeared as two foci that each appeared to migrate to opposite poles at anaphase (Fig. 2C). Drh1p showed localization to both similar fibers and in puncta during Meiosis II as we described for Meiosis I. Overall, this highly organized and dynamic localization of GFP-Drh1p throughout meiosis may reflect association with and/or regulation by the meiotic spindle.

GFP-Drh1p exhibited additional, notable changes in its localization during early conjugation. We did not detect it in the nucleus during the third pre-zygotic division (a mitotic division) (Fig. S4), but did detect it in the nucleus after nuclear exchange and formation of the zygotic nucleus (Fig. 2D, left panel; Fig. S4). During synkaryon formation (the nuclear fusion that produces the zygotic genome), multiple GFP-Drh1p foci appeared to coalesce and segregate during mitotic anaphase. In these zygotic nuclei, we observed GFP-Drh1p as a single large aggregate is some cells, but in others the protein was divided into two or four nuclear foci (Fig. 2D, left panel). This difference may reflect varying degrees of spindle integration that must occur upon nuclear fusion, as individual spindles form prior to karyogamy (Cole and Sugai, 2012).

After karyogamy, the newly formed zygotic nucleus undergoes two rounds of mitosis to produce precursors of the new micro- and macronuclei. Following the second post-zygotic mitosis, the two anterior division products enlarge and begin differentiation into macronuclei. At this stage of development, GFP-Drh1p was in these developing macronuclei and was excluded from the micronuclei. We also observed some transient localization in the parental macronucleus just before its degeneration. It is quite intriguing that Drh1p switches its localization rapidly from micronuclei throughout pre-zygotic development to macronuclei in post-zygotic development (Fig. 2D; Fig. S4). The localization in the differentiating macronuclei was diffuse and did not exhibit the highly organized patterns similar to what we observed in dividing micronuclei. Whether the changing localization reflects different functions for Drh1p in these different nuclear compartments is yet to be determined.

### *DRH1* is essential for growth and development

**Fig. 3.**
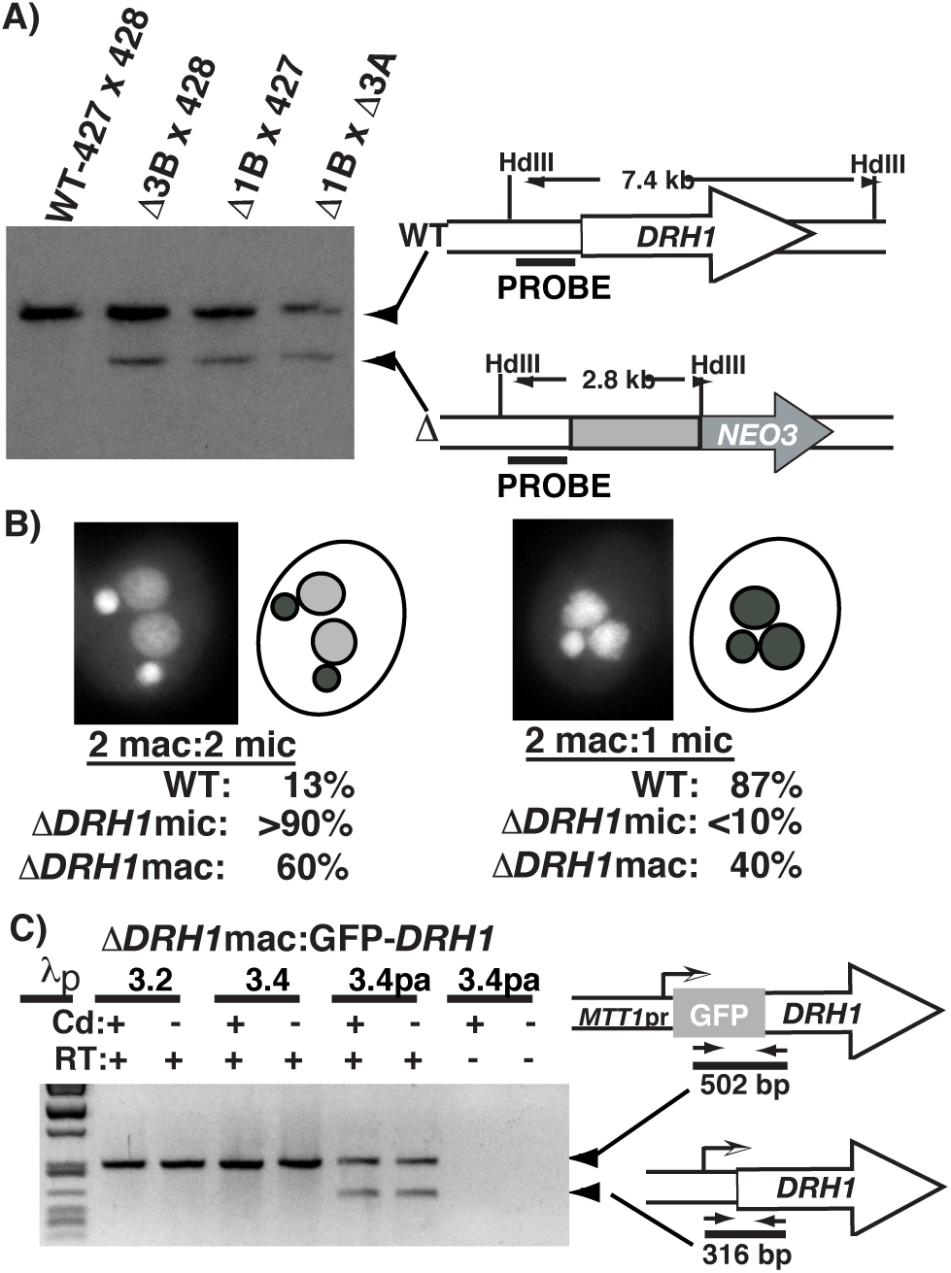
***DRH1* is essential for growth and development.** (A) Germ line and somatic *DRH1* knockout strains were generated by homologous gene replacement with the *NEO3* selection cassette. Southern blot analysis of *Hind*III (HdIII)-digested genomic DNA revealed correct integration of the knockout construct. DNA was isolated after crossing strains to allow easier detection of the knockout allele (Δ) present in the micronuclei of germline knockouts. The knockout allele is shown below the diagram of the wild type (WT). The fragment sizes expected for each allele and region corresponding to the labeled probe are shown. (B) Terminal phenotypes of exconjugants are represented by DAPI-stained images of nuclear configurations that predominant for post-conjugative wild-type (2 mac: 1 mic) and mutant (2 mac: 2 mic) cells. The shading of nuclei in the diagram at the right indicates relative DAPI fluorescence intensity. Percentages of exconjugants with the observed nuclear configurations are given for wild-type, micronuclear knockout and macronuclear knockdown crosses. (C) GFP-*DRH1* expression allows full assortment of the knockout allele in macronuclei. Oligonucleotide primers (short arrows) were used for RT-PCR to distinguish mRNA produced from the wild-type or *MTT1*-GFP-*DRH1* alleles diagrammed to the right. Expression of both alleles is observed in the partially assorted (pa) *ΔDRH1* mac strain 3.4pa whereas only the GFP-*DRH1* allele is observed in *ΔDRH1*mac strains 3.2 and 3.4 indicative of loss of all WT gene copies in macronuclei.

The localization of Drh1p suggested that this protein has important roles in critical nuclear events throughout development. To identify processes in which this protein might act, we disrupted the gene and assessed cells for loss-of-function phenotypes. We generated a gene disruption construct composed of ∼1 kilobase pairs (kbp) DNA fragments corresponding to genomic DNA flanking the *DRH1* coding sequence, cloned on each side of the *neo3* selectable marker. We introduced this *DRH1* knockout cassette into conjugating *Tetrahymena* cells (strains CU428×B2086) by particle bombardment to disrupt gene copies in both the micro- and macronuclei. *DRH1* knockout strains were identified by selection for paromomycin-resistant progeny. Homologous gene replacement was confirmed by PCR (data not shown) and Southern blot analysis (Fig. 3A). The transformed progeny that we initially generated were heterozygous for the knockout allele in their micronuclei (Δ*DRH1*mic/+) and had a significant portion of their *DRH1* macronuclear gene copies disrupted. We also obtained transformants of non-mated cells in the population with only the macronuclear copies disrupted (Δ*DRH1*mac).

*Tetrahymena* macronuclear division is amitotic, allowing random segregation (called assortment) of alleles during growth. Repeated attempts to obtain transformed cells with all macronuclear copies replaced with the knockout allele by sub-cloning transformants into paromomycin-containing growth medium were unsuccessful, indicating that *DRH1* is likely essential for growth. We next generated cells lacking all *DRH1* copies by mating two strains each homozygous in their micronuclei for the knockout allele (Δ*DRH1*mic strains 1B and 3A). This genetic cross offered the possibility of characterizing phenotypes of cells lacking all *DRH1* expression. No viable progeny resulted from this cross. The majority of the 176 mating pairs cloned into growth medium died, and the only viable cells had aborted conjugation without making new macronuclei (Table 1); thus, *DRH1* has essential functions. Crossing either Δ*DRH1*mic strain to wild-type strains rescued the loss of *DRH1* expression from the knockout partner, resulting in a high percentage (64-91%) of viable progeny (Table 1).

**Table 1.**
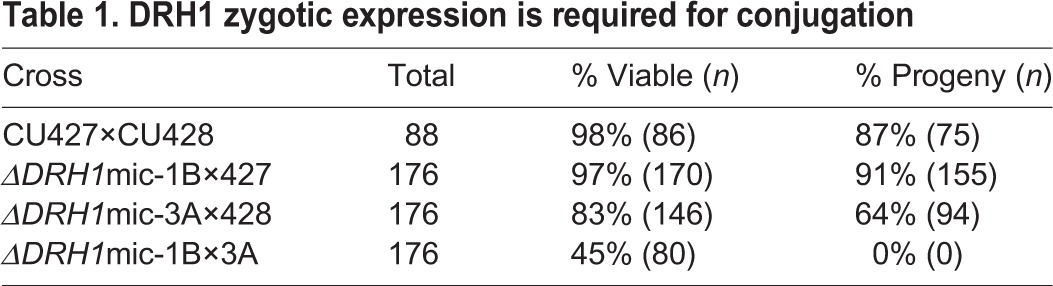
**DRH1 zygotic expression is required for conjugation**

Because the progeny of homozygous Δ*DRH1*mic strains lack all wild-type *DRH1* copies, their failure to survive could have resulted from an inability to (1) grow once all maternally expressed protein was depleted or (2) even complete conjugation without zygotic *DRH1* expression. To first determine whether mated pairs could return to vegetative growth, we isolated individual pairs into growth medium. We observed that these cells either remained paired or, when separated, that the two exconjugants were unable to divide, a phenotype consistent with a developmental arrest. To next determine whether cells arrested before completing conjugation, we fixed cells from mated populations (24-30 h post-mixing without addition of growth medium) and stained their DNA with DAPI because nuclear morphology is diagnostic for specific stages of development (Martindale et al., 1982). The majority of wild-type exconjugants completed mating with one micronucleus and two newly formed macronuclei containing amplified genomes, poised to resume vegetative growth once provided a food source (Fig. 3B; Table 1). In contrast, the mated Δ*DRH1*mic cells arrested prior to the elimination of one micronucleus and, based on the lower intensity of DAPI fluorescence, without fully amplifying the DNA in developing macronuclei (Fig. 3B). We conclude that *DRH1* zygotic expression is required for cells to complete late stages of conjugation, and that *DRH1* is essential for both growth and development.

As the progeny of the Δ*DRH1*mic strains arrested in development, this genetic cross did not allow us to examine the phenotype of loss of *DRH1* function in vegetative cells. In an attempt to overcome this limitation, we generated cells lacking all endogenous *DRH1* copies in macronuclei by expressing an inducible allele of the gene from an ectopic locus. We started with two partially assorted (pa) Δ*DRH1mac* strains into which we introduced a construct consisting of a GFP-*DRH1* fusion expressed from the cadmium inducible *MTT1* promoter and integrated upstream of the *rpl29* locus. By expressing the GFP-*DRH1* fusion allele in these cells, the knockout allele was able to completely replace the endogenous *DRH1* gene, which we demonstrated by using reverse-transcription (RT) PCR in which only mRNA from the GFP-*DRH1* allele was detectable (Fig. 3C). In the absence of all endogenous protein, GFP-Drh1p expressed during vegetative growth localized primarily in the cytoplasm or near basal bodies, supporting the idea that the localization of the GFP-Drh1p expressed from the rDNA vector described above was biologically relevant. To examine the effect of loss-of-function phenotypes in growing cells, we placed the fully assorted Δ*DRH1*mac:GFP-*DRH1* strains into medium lacking cadmium. We found that the cells continued to grow; however, we could detect low levels of GFP-*DRH1* expression by fluorescence microscopy (data not shown) and RT-PCR (Fig. 3C), suggesting that the *MTT1* promoter was too leaky to observe loss-of-function phenotypes in growing cells.

We utilized these Δ*DRH1*mac:GFP-*DRH1* cells to examine loss of *DRH1* expression during early conjugation. These mutant cells, when starved and mated without addition of cadmium, accumulated limited amounts of Drh1p as judged by the low intensity of GFP fluorescence. When these mated pairs were isolated into growth medium, most died and produced no viable progeny. In contrast, crossing these strains to wild-type cells or crossing them together in the presence of cadmium produced viable progeny. Examination of the mated cell population showed that about 60% of the exconjugants arrested with two micro- and two macronuclei, and the remainder appeared to complete conjugation and amplify their new macronuclear genomes, even though no viable progeny resulted (Fig. 3B). This partially penetrant phenotype is consistent with a knockdown, rather than a knockout, phenotype. Nevertheless, the inability of Δ*DRH1*mac:GFP-*DRH1* strains to produce viable progeny demonstrates that expression of *DRH1* from parental macronuclei is required for *Tetrahymena* development.

### *DRH1* zygotic expression is required for chromosome breakage

**Fig. 4.**
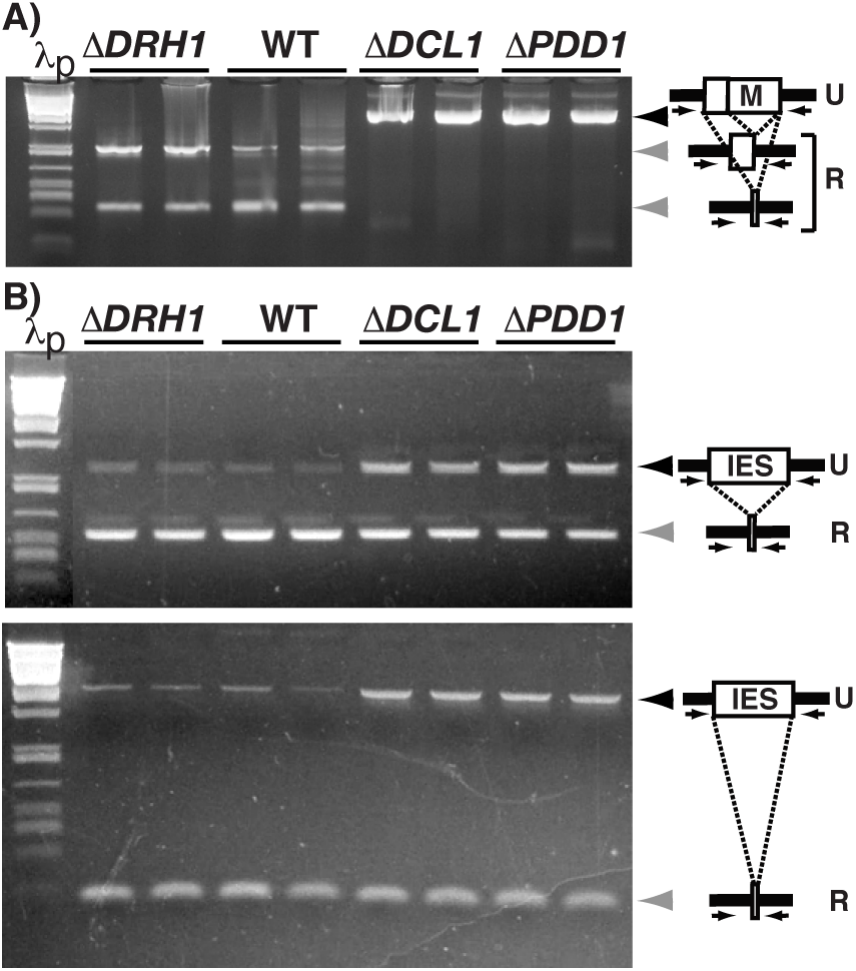
***DRH1* zygotic expression is not required for IES excision.** (A) PCR of single exconjugants from crosses of wild-type (WT) or mutant strains (*ΔDRH1*, *ΔDCL1* and *ΔPDD1*) was performed with primers (small arrows) flanking the M IES. (B) PCR amplification of two additional IESs loci was performed on total DNA isolated from 30-h post-conjugative cultures. Black and gray arrowheads indicate, respectively, the position of migration of unrearranged (U) and rearranged (R) forms of the IES. λp are size standards.

The two micro- and two macronuclei developmental arrest point, which we observed upon mating Δ*DRH1*mic strains, is a common phenotype described for several strains lacking genes required for developmentally programmed DNA rearrangements (Shieh and Chalker, 2013; Rexer and Chalker, 2007; Motl and Chalker, 2011; Malone et al., 2005; Horrell and Chalker, 2014; Noto et al., 2010; Mochizuki et al., 2002; Bednenko et al., 2009). To determine whether the developmental arrest observed upon loss of *DRH1* expression was associated with defects in genome rearrangements, we assessed IES excision and chromosome breakage efficiency in Δ*DRH1*mic strains post-conjugation. We used PCR of single exconjugants to monitor the rearrangement of the well-characterized M IES (Fig. 4A; Table 2) (Godiska et al., 1993; Godiska and Yao, 1990; Chalker and Yao, 1996; Kowalczyk et al., 2006). When this germline-derived locus undergoes DNA elimination, either 0.6 or 0.9 kbp are removed from the developing genome to generate the rearranged forms in the macronuclei of progeny. We detected exclusively the rearranged locus in the 18 exconjugants of Δ*DRH1*mic cells tested (Table 2). This is in striking contrast to control mating of DNA rearrangement mutants Δ*DCL1* or Δ*PDD1*, whose exconjugants fail to eliminate the M IES as indicated by a larger PCR amplification product (Fig. 4A).

**Table 2.**
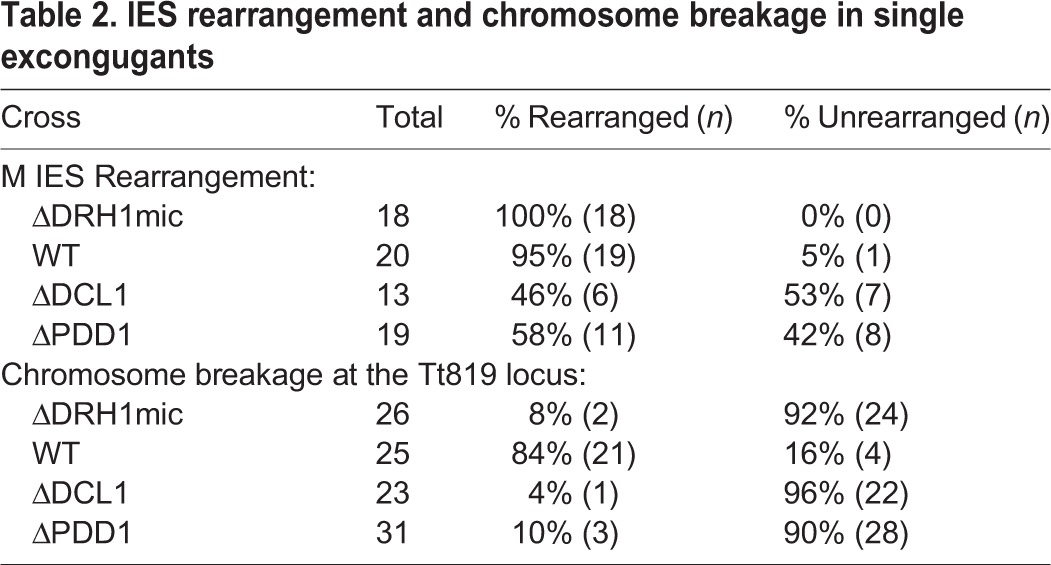
**IES rearrangement and chromosome breakage in single excongugants**

To further characterize DNA rearrangement efficiency in Δ*DRH1*mic exconjugants, we isolated total DNA from populations of mated cells and used PCR (Fig. 4B) or Southern blot analysis (data not shown) to examine the fate of four other IES-containing loci. For each IES examined, the relative abundances of PCR products or DNA fragments that corresponded to unrearranged loci were comparable between wild-type and Δ*DRH1*mic mated populations, whereas these products were over-represented in the analysis of Δ*DCL1* and Δ*PDD1* mated cells, which are mutants unable to excise IESs, when compared to wild type. It is important to note that because we analyzed IES rearrangement in populations of cells, which include unmated cells, we detected the rearranged IESs in all samples. Overall, IES excision was not impaired at any of the five loci examined by loss of *DRH1* zygotic expression.

**Fig. 5.**
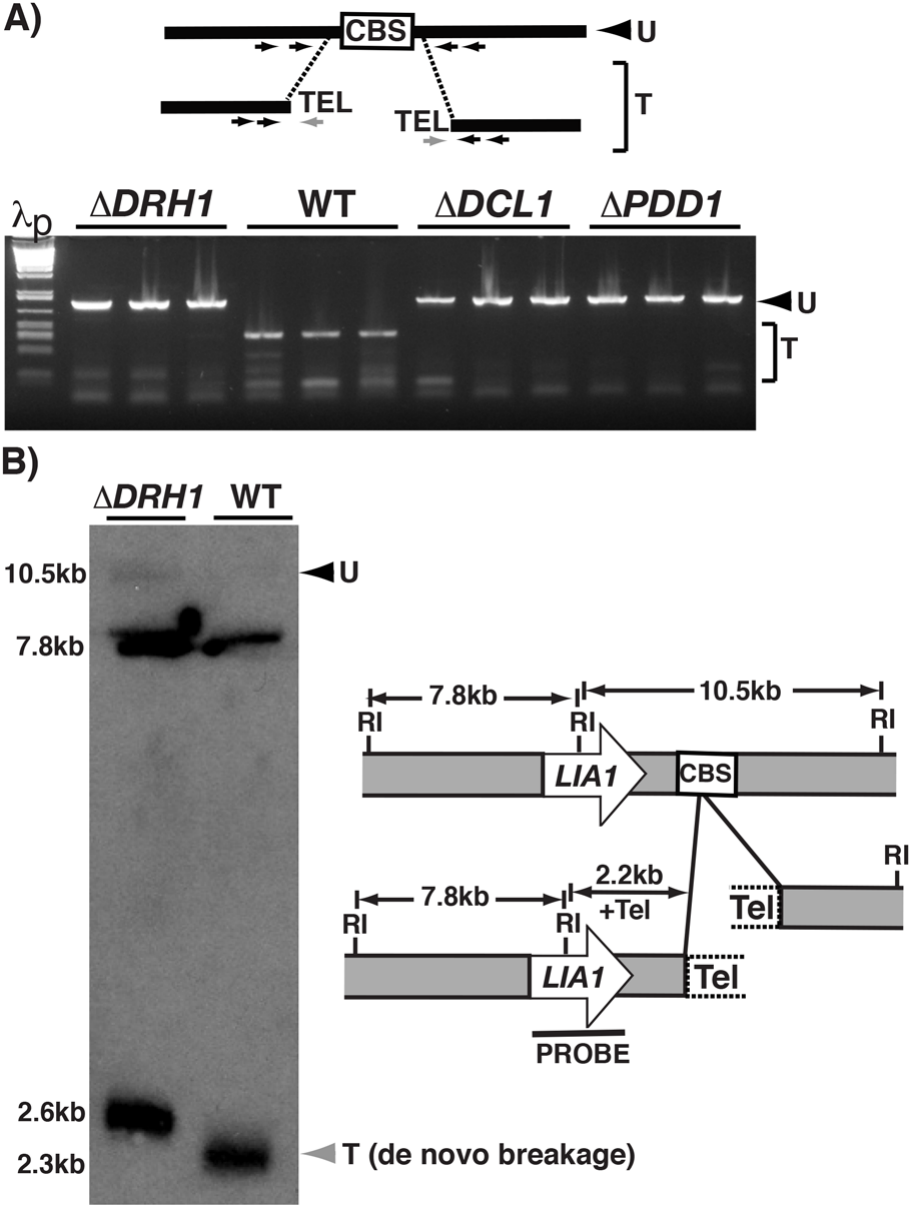
***DRH1* zygotic expression is required for chromosome breakage.** (A) PCR of single exconjugants from crosses of wild-type (WT) or mutant strains (*ΔDRH1*, *ΔDCL1* and *ΔPDD1*) was performed with primers (small arrows) complementary to sequences flanking the Tt819 locus CBS and a telomere (Tel) specific primer that allow detection of both unbroken (U) and telomerized (T) chromosomes. λp are size standards. (B) Southern blot analysis of the telomere-proximal LIA1 locus (depicted on the right) was performed on total DNA isolated from 30-h post-conjugative cultures after digestion with *Eco*RI (RI). Black and gray arrowheads indicate unbroken (U) and telomerized (T) chromosomes, respectively. The sizes of expected products are shown. Telomerized chromosomes from parental macronuclei have elongated chromosomes and thus migrate noticeably higher than chromosomes broken *de novo* during conjugation.

Mutants that fail to perform IES excision typically fail to carry out chromosome breakage, a second DNA rearrangement that couples chromosome fragmentation at specific sites with *de novo* telomere addition. To investigate whether this process was impaired by loss of *DRH1* expression, we examined breakage at the Tt819 locus in single Δ*DRH1*mic exconjugants. We used nested primer pairs flanking Tt819 cbs, which can amplify the unbroken chromosome, together with a telomere-specific primer that amplified the telomerized ends after fragmentation. More than 90% of the exconjugants tested failed to fragment their chromosomes at this locus, results comparable to exconjugants from Δ*DCL1* or Δ*PDD1* crosses (Fig. 5A; Table 2). In other control tests of wild-type exconjugants, the unrearranged locus was only detected in 4 of 25 exconjugants (Table 2), which likely resulted from amplification of the micronuclear copies.

To determine whether other sites of chromosome breakage were affected, we used Southern blot analysis of total DNA isolated from wild type and Δ*DRH1*mic populations post-conjugation to examine fragmentation downstream of the *LIA1* locus (Fig. 5B). In wild-type cells, the appearance of a ∼2.3 kbp terminal fragment (generated by cleavage but with minimally elongated telomeres) and under-representation of the 10.5 kbp unbroken germline locus is indicative of *de novo* chromosome breakage. By contrast in the Δ*DRH1* cell lines, we detected a larger ∼2.6 kbp terminal fragment with fully elongated telomeres (2.6 kbp=2.2 kbp plus 0.4 kbp of telomere sequence) derived from parental macronuclei of the unmated cells remaining in the population and any parental macronuclei not degraded within the mating pairs. Because of the high copy number of macronuclear chromosomes, even a relatively small fraction of unmated cells can result in a significant representation of the parental chromosomes in the DNA analyzed. Additionally, we observed an increased abundance of the 10.5 kbp band corresponding to the unrearranged locus. Thus chromosome breakage was blocked upon loss of zygotic *DRH1* expression at both loci tested. This represents the first identification of a protein that appears to be required for chromosome breakage but does not affect the efficiency of IES excision. This observation further links Drh1p as an essential protein critical for ensuring development of proper chromosome structure.

## DISCUSSION

Our studies of *DRH1* and *LIA2* indicate that they are paralogs related to the p68-family of DExD/H box helicases and have diversified in both expression and function during the *Tetrahymena* evolution. They are best hits in reciprocal blast searches of the *Tetrahymena* genome and the top hits when searching the genome for putative p68 homologs. This is consistent with these both being orthologous to the p68 helicases. *DRH1* expression is essential, but *LIA2* expression is not; thus, like paralogs p68 and p72 in mammalian cells, the proteins encoded by these *Tetrahymena* genes have distinct roles. Drh1p has essential functions during both growth and development and localizes to multiple cellular compartments: near basal bodies during growth, in micronuclei in pre-zygotic development, and in differentiating macronuclei during post-zygotic development, all features that are consistent with the multi-functional role of the p68 family of DEAD box proteins (Fuller-Pace, 2006; Linder, 2006; Cordin et al., 2006).

When we tried to delete *DRH1* from the macronuclear genome, we were unable to obtain cells lacking all copies of the gene from this polygenic somatic nucleus. This observation indicates that Drh1p is essential for growth. We were able to eliminate all copies of the endogenous *DRH1* gene by introducing and expressing an inducible GFP-*DRH1* fusion allele. The fusion protein localized in the cytoplasm and near basal bodies during vegetative development, which suggests that its essential role during growth is outside of the nucleus. When we deleted *DRH1* from the micronuclear genome, we also found that zygotic expression was required for development. Subsequent analyses revealed the role of Drh1p in fragmentating developing somatic chromosomes. It was striking that we did not detect defects in the scnRNA-dependent IES excision pathway, a finding that makes Drh1p the first protein specifically required for chromosome breakage/*de novo* telomere addition during macronuclear differentiation.

The temporal and mechanistic relationships between internal DNA elimination and chromosome fragmentation remain to be elucidated. Cells with deletions of genes involved in the IES excision pathway, including *DCL1* and *PDD1*, exhibit failure of chromosome breakage, but otherwise, these two processes are not mechanistically linked. The strains lacking genes critical for IES excision arrest in development near the time period when chromosome breakage occurs, so it is plausible that the arrest associated with failed IES excision stops chromosome breakage from occurring. Δ*DRH1*mic cells that lack *DRH1* expression from developing macronuclei also arrested late in development (Fig. 3B), but IESs were excised normally and only chromosome breakage was blocked (Figs 4 and 5). Clearly, these two chromosomal rearrangement events have separate requirements, and Drh1p is specifically required for chromosome breakage.

We have yet to determine whether Drh1p acts directly at sites of chromosome breakage or is more indirectly involved by facilitating the assembly of RNA-protein complexes that are responsible for fragmentation. The protein's localization within meiotic micronuclei suggests a direct role in interacting with chromosomes. In prophase, Drh1p localized to the regions of the elongated nucleus where centromeres and telomeres are positioned (Loidl and Scherthan, 2004; Cervantes et al., 2006). Later in pre-zygotic development, Drh1p localized in foci and fibers, possibly with the spindle apparatus. The mouse p68 helicase binds to satellite DNA and also localizes to fibers and in puncta in the nucleus (Gavrilova et al., 2009). These similarities lead us to suggest that the interaction of p68 proteins with chromosomes has been conserved through these proteins' evolution and indicate that they possess a fundamental role in chromosome structure and/or maintenance.

Because *DRH1* was essential for growth, and the inducible GFP-*DRH1* we introduced was too leaky to abrogate expression, even without induction, we could not determine how absence of Drh1p affected meiotic chromosome behavior. It remains to be determined whether the mechanism by which Drh1p interacts with meiotic chromosomes is similar to its role in chromosome fragmentation. At this point, we can only speculate on the possible roles of this RNA helicase in fragmentation. In *Drosophila melanogaster*, the P68 RNA helicase promotes release of RNA transcripts from chromatin (Buszczak and Spradling, 2006). There is no direct evidence to date that RNAs or transcription are important for chromosome fragmentation, but our discovery of a role for this putative RNA helicase in this process provides new experimental directions to pursue.

## MATERIALS AND METHODS

### Stocks and culture conditions

Wild-type laboratory strains B2086 (MTII), CU428 [*Mpr1-1/Mpr1-1* (VII, mp-s)], and CU427 [*Chx1-1/Chx1-1* (VI, cyc-s)] were used to generate all strains used in this study. The strains were originally obtained from Peter J. Bruns (Cornell University, Ithaca, NY) and are available from the *Tetrahymena* Stock Center (https://tetrahymena.vet.cornell.edu). These strains were also used to confirm progeny production and fertility of the mutant strains. All cells were grown in either 1×Neff's medium or 1×SPP at 30°C. For matings, cells were starved >6 h in 10 mM Tris-HCl (pH 7.4). Complementary mating types were then mixed in equal numbers.

### Drh1p localization

The coding region of *DRH1* was amplified from genomic DNA by PCR using primers [5′ primer ACCTCGAGATGTCAAGATAAATCTAAAGCAATTCT (the *Xho*1 site is underlined) and 3′ primer TGGGCCCTCAGTTGTCTTTCTTTGGGTTG (the *Apa*1 site is underlined)] that added an *Xho*I site upstream of the ATG start codon and an *Apa*I site downstream of the stop codon. The amplified fragment was initially cloned into pCR2.1 using topoisomerase mediated cloning (Invitrogen/Life Technologies, Carlsbad, CA). The resulting plasmid was digested with *Xho*I and *Apa*I, and the amplified coding sequence was inserted into the *Xho*I and *Apa*I sites of pIGF-1 (Malone et al., 2005). The pIGF-DRH1 plasmid was electroporated into conjugating CU427 and CU428 cell as described (Gaertig and Gorovsky, 1995; Gaertig et al., 1994).

### Cadmium induction and confocal microscopy

Wild-type *Tetrahymena* strains CU428 and SB1969 and pIGF-*DRH1* transformed strain A1 were grown to log phase in Neff's medium. Cells were washed in 10 mM Tris (pH 7.4) and GFP-DRH1 expression was induced by addition of 0.2 mg/ml CdCl_2_ and incubating cells for 3 h at 30°C. Cells were then washed, starved (11-12 h at 30°C), and mixed in 10 mM Tris medium. SB1969 cells were mixed with GFP-*DRH1* strain A1, and live cells were viewed with upright epifluorescence and/or confocal microscopy between 2 and 10 h after mixing. For standard epifluorescence microscopy, 0.5 ml of cells were harvested by low-speed centrifugation (1000×***g***) and immobilized in ∼6 µl of 2% methylcellulose. Differential interference contrast and GFP fluorescence images were captured by using a Qimaging RetigaEX charge-coupled-device camera (Burnaby, BC, Canada) and Openlab software (PerkinElmer). Confocal microscopy was performed with an Olympus BX-50 microscope equipped with a Fluoview SV300 scanning laser confocal imaging system. Images were cropped, and their brightness and contrast were adjusted uniformly when necessary in Adobe Photoshop CS5.

### Gene disruption of *LIA2* and *DRH1*

Biolistic transformation was used to integrate *Neo3* (*MTT1-Neo*) (Shang et al., 2002) into the micro- and macronuclear genomes in place of the conserved DEAD box helicase domain of *LIA2*. A 1.5 kbp upstream region of the *LIA2* locus was amplified from *Tetrahymena* genomic DNA by using PCR with oligonucleotides 5′-SacI-Lia2-39 (CGAGCTCAGCCAAATCACCTCATGG) and 3′-NotI-Lia2-434r (ATAAGAATGCGGCCGCTCTTTAGGAGTATTTGTCAGC), then cloned into the *Not*I and *Sac*I sites of pMNBL. Likewise, a 0.87 kbp downstream region was amplified by using PCR with oligonucleotides 5′-XhoI-Lia2-1097 (CCGCTCGAGCTGGAGCATATGGTTGTGCAG) and 3′-ApaI-Lia2-1562r (CCGGGCCCtcaagaagatgttgttgtatt), then cloned into the *Apa*1 and *Xho*I sites of pMNBL containing the upstream *LIA2* fragment to generate pLIA2-KO.

To generate *DRH1* macronuclear and micronuclear knockout strains, the MultiSite Gateway cloning kit (Invitrogen) was used. Sequences upstream (amplified using primers P68_MS5′upAVR GGGGACAACTTTGTATAGAAAAGTTGCCTAGGCTGCTTATGTTGCCTTG and P68_MS5′downAVR_2 GGGGACTGCTTTTTTGTACAAACTTGCCTTCTCCCTTATTAGAATTGC) and downstream (amplified using primers P68_MS3′upAVR GGGACAGCTTTCTGTACAAAGTGGTCGGTTCTTTCAACCCAAAG and P68_MS3′downAVR GGGGACAACTTTGTATAATAAAGTTGCCTAGGATCGTTTATTAATGGAAGGGTCT) of the *DRH1* (Ttherm_00190830) were amplified and incorporated into the pDONR-P4-P1 and pDONR-P2-R3 vectors, respectively, with BP recombinase. pENTR-*MTT1*-Neo (Motl and Chalker, 2011) and the two homology vectors were mixed in equal molar ratios in the presence of the destination vector pDEST-R40-R3 and LR Clonase Plus to create the Ttp68(*DRH1*)-knockout vector.

Plasmid pLIA2-KO was digested with *Apa*I and *Sac*I, and plasmid pTtp68-KO was digested with *Avr*II. The two linearized plasmids were then transformed by biolistics into a mating population of B2086 and CU428 cells 2 h post-mixing as described (Bruns and Cassidy-Hanley, 2000; Cassidy-Hanley et al., 1997). Macronuclear and micronuclear transformants were selected by growth in the presence of paromomycin (80 μg/ml) and CdCl_2_ (1 μg/ml). Micronuclear transformants were further selected for by growth in 6-methylpurine. Micronuclear transformants were examined for progeny production by mating them with CU427 cells and were tested against both Cd/paromomycin and cyclohexamide. The heterozygous micronuclear knockout strain was mated to the star strain B*VII. The compatible ex-conjugant was then mated to B*VI to create homozygous micronuclear knockout strains. Micronuclear strains were confirmed to have insertions in the correct location via Southern blot. The 5′ homology from the knockout vector was used as a probe. Genomic DNA isolated with the Promega Genomic DNA Isolation Kit and was digested with *Hind*III.

### Creating fully assorted macronuclear knockouts

The entire coding region of *DRH1* was amplified by using Phusion High Fidelity Polymerase and the primers p68_up-GTW (CACCTATCAAATGTCAAGATAAATCTAAAGC) and p68_downRV (GATATCAGTTGTCTTTCTTTGGGTTG) and cloned into pENTR/D by Topoisomerase mediated cloning (Invitrogen/Life Technologies). The product was then recombined into the pBS_MTTGFPGTW vector with LR Clonase Plus. The vector was linearized with *Hind*III and biolistically introduced into both of the macronuclear knockout strains. Successful transformants were selected for by growth in media containing both paromomycin and cyclohexamide. Fertile strains were selected and subsequently subcloned until full macronuclear assortment had occurred. Assortment was monitored by using a three-primer PCR assay. The primers were: p68_140r (TTTTCCTTTGGATAGCTTAGACA), p68_-198fV2 (TGCACAGACGAGAATTTTGAA), and Die5NeoUPRV (GGAGTTATTCAAAACCCTTATTATTTT). Full assortment was confirmed via RT-PCR with the following primers: p68_30 (CAAAAACTAATAACCAAATAATTATATC), p68_617r (CTTTTTATTGCATTGAGTATCC), and GFP_566 (GATGGCCCTGTCCTTTTA).

### IES excision tests

The M-element single-cell PCR assays were performed via a nested PCR reaction at 30 h post mixing. Round one primers were M002 and M1201RC. Round two primers were M1194 and M110. The CaM locus was examined via Southern blot. A small fragment upstream of the CaM gene was used as a probe. Total genomic DNA was isolated 30+ hours post mixing and digested with EcoRI. IES1, IES7, and IES11 were all assayed in total genomic DNA isolated at 30+ hours post mixing with the following primer sets: IES1_MDSL-110 (TGAAGATCTACTTCAAAGCGAAT) and IES1_MDSR-31 (CCAGCTAGACACCCTGTATCAA), IES7_MDSL-112 (GGATGATTGCATAAATGGA) and IES7_MDSR-158 (ACCCAGAATACCGCAGTTC), IES11_MDSL-42 (GGCCACAATATACTAAGGCAATTT) and IES11_MDSR (GGCCACGTTGATACCAGTTT).

### Chromosome breakage tests

The chromosome breakage site downstream of the *LIA1* gene was analyzed via Southern blot. Total genomic DNA was isolated 30+ hours post mixing and digested with *Eco*RI. The Tt819 locus was examined by single-cell PCR. This was performed as a nested PCR reaction on cells 30+ hours post mixing. Round one used the following primers: Tt819-1 (GATCAAACTGAGACTCACTATC), Tt819-3 (GATCAATTCATTTTAATTAATTTAG), and Tel1 (CCCCAACCCCAACCCCAA). Round two used the following primers: Tt819-2 (TCAAAACTTATCCAGGATTAAAG), Tt819-4 (ATTTTATTAGTTATCTTTTAGTAAAG), and Tel1 (CCCCAACCCCAACCCCAA).
